# Animal Models of GWAS-Identified Type 2 Diabetes Genes

**DOI:** 10.1155/2013/906590

**Published:** 2013-04-11

**Authors:** Gabriela da Silva Xavier, Elisa A. Bellomo, James A. McGinty, Paul M. French, Guy A. Rutter

**Affiliations:** ^1^Section of Cell Biology, Division of Diabetes, Endocrinology and Metabolism, Department of Medicine, Imperial College London, London SW7 2AZ, UK; ^2^Biophotonics Section, Department of Physics, Imperial College London, London SW7 2AZ, UK

## Abstract

More than 65 *loci*, encoding up to 500 different genes, have been implicated by genome-wide association studies (GWAS) as conferring an increased risk of developing type 2 diabetes (T2D). Whilst mouse models have in the past been central to understanding the mechanisms through which more penetrant risk genes for T2D, for example, those responsible for neonatal or maturity-onset diabetes of the young, only a few of those identified by GWAS, notably *TCF7L2* and *ZnT8/SLC30A8*, have to date been examined in mouse models. We discuss here the animal models available for the latter genes and provide perspectives for future, higher throughput approaches towards efficiently mining the information provided by human genetics.

## 1. Introduction

The estimated global prevalence for diabetes in 2011 was 366 million, and the disease is expected to affect 552 million people by 2030 (Diabetes U.K. figures; [1] accessed 09/01/13). Type 2 diabetes (T2D) is a complex and multifactorial disease characterised by impaired insulin secretion and insulin resistance. Disease risk/progression is determined by a combination of genetic and environmental factors. It has been consistently demonstrated that lifestyle factors are associated with risk of T2D across populations [2–8], with increased adiposity being the greatest modifiable risk factor for the disease [9, 10]. Inactivity [3, 11], “bad” diet [2, 6, 8, 12–14], smoking, and other vices [8, 15, 16] and the nutritional environment during pre- and postnatal life [17] also contribute to the risk for developing diabetes. 

It has been estimated that 30–70% of  T2D risk may be due to genetics [18]. Whilst pedigree-based linkage analysis and the candidate gene approach led to the discovery of highly penetrant genetic defects which account for the development of diabetes [19–24], it is the advent of large scale genome-wide association studies (GWAS) which have led to the accelerated discovery of risk-variants associated with T2D [25–34]. Currently, over 60 common risk variants have been identified [30–34], with a combined disease risk of 5–10% [34, 35], suggesting the existence of many more as yet undiscovered loci [34, 36, 37]. Most of the GWAS-identified associations for T2D have high linkage disequilibrium with a causal variant with a small effect size; the largest common variant-signal identified to date is that for *TCF7L2,* which has a per allelle odds ratio of 1.35 [27–29].

Most of the common variant signals identified by GWAS are associated with defective pancreatic islet function, indicating that this is the primary driver for the development of T2D [34, 38]. However, most of the GWAS signals map to noncoding regions of the genome, making it difficult to establish functional links to specific transcripts. As a result, determination both of (a) the identity of the likely transcript(s) involved and (b) the mechanisms of actions on disease risk, require the use of genetically tractable organisms where the expression of candidate genes can be manipulated at will in a cell type-specific manner. Of the available models (which include lower organisms such as *C. elegans*, *D. melanogaster,* etc.), mice arguably represent the best compromise between ease of genetic manipulation and similarity to man, in terms of both genome structure and physiology. In this review, we discuss the use of mouse models to study the contribution of genetic variations, identified by GWAS, in the *TCF7L2* and *SLC30A8* genes to the development of T2D via their effects on pancreatic islet function.

## 2. *TCF7L2*

### 2.1. Background

The gene-encoding Transcription 7 Like-2 (*TCF7L2*, previously called TCF4) is the most important T2D susceptibility gene identified to date, with genetic variants strongly associated with diabetes in all major racial groups [27–29, 39–59]. Signals in this locus are the most consistently identified across various GWAS and are associated with the highest elevation of risk of developing adult-onset T2D. Each copy of the risk *T-*allele at rs7903146 has an increased odds ratio for T2D of 1.4-1.5 [60]. Inheritance of the risk allele is also a useful predictor for the likelihood of conversion from a state of prediabetes to T2D [61, 62]. Additionally, results from a small number of studies also indicate that *TCF7L2* variation may play an important role in cases of early onset T2D [63, 64].

*TCF7L2* is a member of the TCF family of transcription factors involved in the control of cell growth and signalling downstream of wingless-type MMTV integration site family (Wnt) receptors [65]. Activation of the Wnt pathway leads to release of *β*-catenin from an inhibitory complex and its translocation to the nucleus, where it binds *TCF7L2* and other related TCF factors [66]. The function of this transcriptional complex is context dependent; that is it may act as either a transcriptional activator or repressor [66].

In recent years, the product of the *TCF7L2 * gene has been associated with dysregulated pancreatic *β* cell function and T2D [25, 27, 28]. Enhanced Wnt signalling has been shown to lead to proliferation of islets [67] and the pancreatic epithelium [68]. Whilst loss of *β*-catenin signalling has been shown to lead to pancreatic hypoplasia [69], stabilisation of *β*-catenin has been shown to result in the formation of large pancreatic tumours [70].

Individuals carrying the risk alleles of rs7903146 in the *TCF7L2* gene display lowered insulin secretion [61, 71, 72], impaired insulin processing [71], and decreased sensitivity to the incretin glucagon-like peptide 1 (GLP-1) [72, 73] compared to controls. *TCF7L2* message levels were elevated in T2D patients [72, 74], whilst *TCF7L2* protein content was depressed [75]. The decrease in protein content was associated with downregulation of GLP-1 and gastric inhibitory peptide (GIP) receptor expression and impaired pancreatic *β* cell function [74, 76, 77]. Studies have shown that silencing of *Tcf7l2* gene expression in clonal mouse *β* cell lines [76] and primary islets [75] leads to increased apoptosis [75] and impaired *β* cell function [19, 20]. Gene expression analysis following *Tcf7l2 *silencing revealed changes in the expression of a number of genes in mouse pancreatic islets [76], one of which was *Glp1r* [73, 78]. *TCF7L2* may mediate GLP-1-induced *β* cell proliferation through activation of the Wnt signalling pathway [79]. Since GLP-1 is implicated in *β* cell survival, the increased incidence of apoptosis in *TCF7L2*-silenced islets [74, 75] and in individuals carrying the variants of *TCF7L2 *[73] is consistent with lowered GLP-1 signalling [73, 78]. Correspondingly, the diminished insulinotropic effect of GLP-1 in *Tcf7l2*-silenced islets may be due, at least in part, to the lack of cognate receptors on the cell surface [74].

### 2.2. Mouse Models for *TCF7L2*

#### 2.2.1. Whole Body Knockout Model

Prior to its association with T2D, *TCF7L2* was previously best known for its association with cancer development [80–82]. Homozygous *Tcf7l2* knockout (*Tcf7l2* ^−/−^) mice die shortly after birth, with a lack of stem cells in their intestinal crypts [83]. Newborn* Tcf7l2* ^−/−^ mice have reduced body weight with significantly lower blood glucose 3 h postpartum than control littermates, which is not caused by excessive insulin release but by impaired carbohydrate and lipid metabolism in the newborn liver [84].

Heterozygote *Tcf7l2* ^+/−^ mice display >20% decrease in body weight compared to wild-type littermates, with decreased glucose, insulin, fatty acid, triglyceride, and cholesterol in adult mice [84]. *Tcf7l2* ^+/−^ mice displayed increased insulin sensitivity, improved glucose tolerance, and reduced hepatic glucose output [84, 85]. Improved glucose tolerance was also observed in heterozygote null mice generated using zinc finger nucleases [85] and insertion of a loxP site and FRT-flanked neomycin selection cassette within intron 4 and a loxP site within intron 5 [86], with data from the latter study also pointing to reduced lipogenesis and hepatic triglyceride levels and decreased peripheral fat deposition following exposure to a high fat diet in heterozygote mice compared to control littermates.

Pancreatic development is grossly normal in *Tcf7l2* ^−/−^ mice [83, 84]. This observation and a report suggesting that *TCF7L2* was not expressed in the pancreas [87] led to the proposal that the principle defect underlying decreased insulin production in TC- or TT-bearing individuals may be inadequate production of GLP-1, from gut L-cells [88]. However, evidence for differences in GLP-1 level in individuals with the common *versus* the at-risk *TCF7L2* allele is currently absent [89], and patient studies have indicated that the primary defect lies in pancreatic *β* cells [71, 72, 75]. For this reason, mouse models which allow *Tcf7l2* gene expression to be selectively ablated in the islet were required.

#### 2.2.2. Pancreas Knockout Model

We used the *Pdx1* promoter-driven Cre recombinase (PDX1.*Cre)* deleter strain [90] to effect deletion in all cells of pancreatic lineage in transgenic mice with a floxed *Tcf7l2* exon 1 to address the question whether selective deletion of *Tcf7l2* in pancreas impairs or improves glucose homeostasis and insulin secretion [77]. This approach allowed us to detect the potential effects of *Tcf7l2* deletion early in pancreatic development, as *TCF7L2* has previously been shown to regulate cell proliferation during development: the *Tcf7l2* ^−/−^ mouse exhibited defects in the accumulation of stem cells in the intestinal crypt [83]. This approach also offered an advantage over the use of the commonly deployed rat insulin 2-promoter-driven Cre recombinase (RIP2.*Cre*) deleter strain since the latter also leads to deletion in the central nervous system [91–93]. Pancreas-specific *Tcf7l2* ^−/−^ (pTcf7l2) mice showed age-dependent glucose intolerance by 20 weeks of age when challenged with an intraperitoneal glucose bolus [77]. Glucose intolerance was detected from 12 weeks of age when glucose was administered by the oral route, indicating that the incretin response was impaired [77]. Tolerance to glucose introduced by both the oral and intraperitoneal route was exacerbated in pTcf7l2 mice that were exposed to a high fat diet, with a concomitant decrease in *β* cell mass [77]. The latter observation is consistent with observations by Shu and colleagues in high-fat-fed rats [94], where the authors found a correlation of *Tcf7l2* expression and *β* cell regeneration from pancreatic ductal cells and may reflect the inability of *β* cells to proliferate or regenerate from progenitor cells in the absence of functional *Tcf7l2*. The decreased expression of the *cyclin D1* gene [77] from islets of Langerhans extracted from 20-week-old pTcf7l2 mice may contribute towards the lack of cell proliferation.

*pTcf7l2* ^−/−^ islets displayed impaired glucose and GLP-1-stimulated insulin secretion and decreased expression of the gene encoding for the GLP-1 receptor [77], consistent with *in vitro* human and mouse islet and cell line siRNA-mediated-silencing experiments [74–76]. Whilst the PDX1.*Cre* strain is likely to result in deletion in other (non-*β*) cell types [95], we observed no difference in plasma glucagon and GLP-1 levels and in insulin sensitivity in pTcf7l2 mice [77]. Our preliminary data obtained using a more *β* cell selective deleter strain (Ins1.*Cre*; J. Ferrer, B. Thorens, unpublished) also indicate deficiencies in insulin secretion and glucose tolerance, suggesting that *TCF7L2* plays a critical and cell autonomous role in the *β* cell compartment.

#### 2.2.3. *β* Cell Knockout Model

Recently, Boj and colleagues generated a *β* cell *Tcf7l2* knockout (*β*TCF4KO) mouse using the tamoxifen inducible RIP2.*Cre*-ER^T2^ deleter strain [96] bred against a conditional mouse-bearing *Tcf7l2* alleles with a *flox*ed exon 10 [84]. Although the use of the RIP2.Cre-ER^T2^ may affect metabolic phenotype through the expression of *Cre* recombinase in islet cells as well as in hypothalamic neurons [95], it is unclear whether *Tcf7l2* expression was affected in the hypothalamus of *β*TCF4KO mice.

*β*TCF4KO mice on normal or high fat diet displayed normal glucose tolerance when glucose was introduced by the intraperitoneal route [84]. There was no difference in plasma insulin and insulin release from isolated islets of *β*TCF4KO mice, *versus* control littermates, in response to glucose challenge [84]. Importantly, however, mice were not examined beyond 12 weeks of age, and oral glucose tolerances were not reported in this later study.

#### 2.2.4. Transgenic Models

In the three previous mouse models described in this section, *Tcf7l2* gene expression was ablated either constitutively [83] or specifically in islet cells [77, 84]. Savic and colleagues took a different approach whereby they engineered mice that expressed LacZ under the control of human bacterial artificial chromosomes (BACs) containing the genomic interval encompassing the diabetes associated SNPs (which are intronic) for *TCF7L2 *[85]. Using this technique, they demonstrated the presence of enhancer function in the SNPs-containing region which drives expression in, for example, intestine and pancreas, but not in adult islets [85]. Transgenic mice with *Tcf7l2* overexpression driven by the human BAC sequence exhibited glucose intolerance when placed on a high fat diet [85]. These data are consistent with that presented by Gaulton and colleagues [103] indicating that the chromatin of the *TCF7L2* intronic variant is in an islet-specific “open” conformation, and reporter assays demonstrated increased enhancer activity of the at-risk *T-*allelle compared with the *C-*allelle in *β* cell lines.

The discrepancy in data between the various mouse models could be partly due to the involvement of *TCF7L2* in glucose homeostasis in more than one tissue, and at different times during development. The *Tcf7l2* gene was manipulated in different ways in the various experimental models and this may alter the tissue-specific splicing of the gene [104–107]. The expression of different variants may lead to different outcomes in different tissue types [104–107]. Analysis of glucose homeostasis at different time points during the life time of the animals and exposure to differing amounts of time to diets with different fat composition could all contribute to the differences in observations.

## 3. ZnT8 (SLC30A8)

### 3.1. Background

ZnT8 (encoding by the *SLC30A8* gene) is a member of the zinc transporter family (ZnTs) important for extruding zinc from the cytosol into either the extracellular space or intracellular organelles [108]. In particular, the expression of ZnT8 is largely (but not exclusively) restricted to *α* and *β* cells of the islets of Langerhans, where the mature protein resides chiefly on the limiting membrane of dense core secretory granule [97, 109, 110]. Its function thus, appears to be chiefly to transport Zn^2+^ from the cytosol into the granules where, in beta cells, this is required for insulin crystallisation [111]. By contrast, the role of Zn^2+^ in glucagon storage in the pancreatic alpha cell granule is not fully understood. 

From the discovery that a single nucleotide polymorphism in the *SLC30A8* gene leads to an increased risk of developing T2D [27, 29], much work has been done to elucidate the function of the encoded protein and the role that ZnT8 plays in the pathogenesis of the disease. In contrast with the majority of  GWAS-identified polymorphisms, rs13266634 in the *SLC30A8* gene encodes the replacement of  Trp for Arg at position 325 (R325W) at the C-terminus of the protein and is associated with a ~20% increased risk of developing T2D per allele [28]. Given the highly restricted expression pattern of the transporter, hopes have been raised that ZnT8 may provide an exciting new drug target to enhance insulin release in diabetic patients.

### 3.2. Mouse Models Exploring *ZnT8/SLC30A8* Function

**Figure 1:**
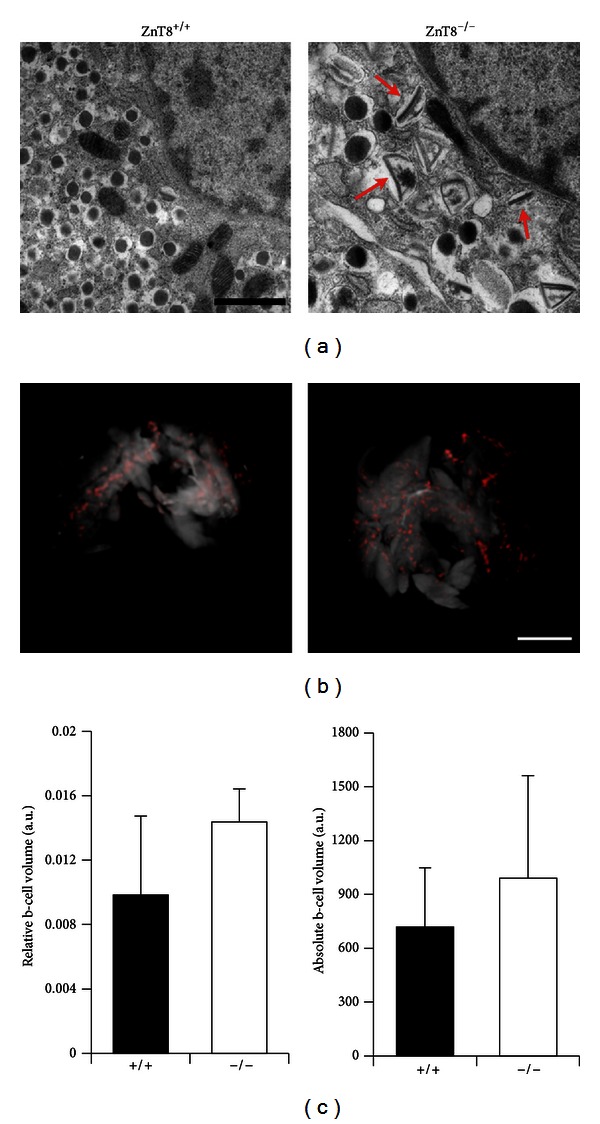
Electron Micrographs and Optical Projection Tomography (OPT) in ZnT8^+/+^ and ZnT8^−/−^ mice. (a) Transmission electron microscopy images of isolated islets from ZnT8^+/+^ and ZnT8^−/−^ male mice at high magnification (scale bar 1 mm) reveals the appearance of rod-shaped core granules in ZnT8^−/−^ cells, indicated by red arrows (*n* = 3 mice). Sections were cut and images were acquired by Dr. Raffaella Carzaniga and Ms. Katrin Kronenberger. (b) Representative three-dimensional OPT projections of whole fixed and permeabilised pancreas from ZnT8^−/−^ and ZnT8^+/+^ mice. In red are the insulin positive structures (*β* cells). The overall shape of the whole pancreas was visualized as autofluorescence and is apparent as white/grey shading. Scale bar = 1 cm. (c) Relative (right panel) and absolute (left panel) *β*-cell volume (*n* = 2 pancreata per genotype).

A number of mouse models have been generated in order to elucidate the function of this molecule and its role in the pathogenesis of diabetes. These include whole body [97, 99–101] and cell type-specific (*α* or *β* cell) [102] ZnT8 knockout animals. Systemic ZnT8 knockout models have up to now been investigated by three different groups [97, 99–101]. These studies have revealed gross abnormalities (albeit age and gender dependent) in insulin crystallisation and storage [97, 99] (Figure 1(a)), confirming the importance of ZnT8 in granular zinc accumulation. Nonetheless, significant differences were apparent both in terms of the regulation of insulin secretion and whole body glucose homeostasis. These differences are likely the result of subtle differences in genetic background, gender, and the age of the animals. Local environmental factors including diet and gut microbiome may also play a role [112]. Thus, glucose tolerance was found to be impaired at an early age (4–6 weeks of age) in three of these studies but not at an older age (>18 weeks) [97, 99, 100], suggesting that the penetrance of the phenotype decreases with age. While insulin sensitivity was unaltered in all of the studies, defects in insulin secretion were reported in two of the studies [97, 100]. None of these changes was associated with altered beta cell mass (Figures 1(b) and 1(c)) These data support the view that decreased ZnT8 activity is likely to influence glucose homeostasis in man and may underlie the defects which increase the risk of developing T2D. Differences in the phenotype are summarised in Table 1.

Of note, the two recent studies of Pound et al. stress the importance of the genetic background. In the first [100], the genetically modified animals were maintained on a mixed background, while in the second [101], the mice were backcrossed onto a pure C57BL/6 background. Strikingly, whereas glucose-stimulated insulin secretion was unaltered in islets from ZnT8 knockout mice on a pure C57BL/6 background, islets from mice on the mixed background showed clear abnormalities in this parameter. Again, plasma insulin was decreased in the mixed background animals, while it was found normal in mice on a pure background. Since these mice were generated and kept in the same animal facility, it seems reasonable to exclude environmental differences as playing a role. Instead, these data support the view that background is a critical determinant of the penetrance of null ZnT8 alleles. Whether this impacts the preservation of functional *β* cell mass in the face of differing insulin sensitivities between the strains, altered intracellular Zn^2+^ handling, or defective auto/paracrine Zn^2+^ signalling between islets cells remains to be elucidated.

As mentioned above, ZnT8 is present in both *α* and *β* cells such that the systemic knockout model reflects the impact of deletion from both cell types (and perhaps others where ZnT8 is expressed at low but detectable levels). The generation of cell-specific knockout models has therefore helped in understanding the contribution of each cell type to the overall phenotype observed. Wijesekara et al. described both the animal models in a recent paper [102]. Deletion of ZnT8 selectively from *β* cells (*β*ZnT8 null mice; using the RIP2 promoter) led to similar effects on glucose homeostasis as those observed in the systemic knockout developed by the same group and by ourselves [97], confirming that the transporter is required for proper insulin processing, crystallization, and packaging. However, *β*ZnT8 null mice displayed additional abnormalities in the expression of key genes required for normal glucose sensing in *β* cells. Whilst the underlying reasons for this greater penetrance are unclear, it is possible that changes in intracellular free Zn^2+^ levels are more marked in the *β* cell selective model since the *α* cell complement remains as an efficient sink for Zn^2+^ release, thus, more efficiently depleting *β* cell Zn^2+^. Nonetheless, the broad similarities between ZnT8 whole body knockout and the *β*-cell-specific mouse model suggest that the phenotype of the former is primarily a consequence of ZnT8 deletion in *β* and not *α* cells; *α* cell-selective ZnT8 null mouse displayed unaltered glucose tolerance. However, glucagon secretion was not measured in these animals under conditions where the latter is likely to be physiologically important that is hypoglycaemia. It therefore remains possible that ZnT8 plays a significant role in the *α* cell, a possibility which awaits more detailed examination of *α* cell-selective null mice in the future.

Because T2D is a polygenic disease that is also influenced by environmental factors, it is important to mention new studies where ZnT8 knockout animals were maintained on a high fat content diet (HFD) [98, 99]. In both of these studies, ZnT8 null mice displayed an increase in body weight as well as fasting blood glucose and insulin levels compared to wild type controls. In particular, in one of these studies, 50% of the ZnT8 knockout animals became hyperglycemic after exposure to HFD, while none of the controls did so [99]. Each of these studies was performed using animals on a mixed (sv129/C57BL6) background. On the other hand, a further recent study using animals backcrossed onto a C57BL6 background [101] revealed that ZnT8 null animals were protected against the effects of high fat, again stressing the likely importance of modifier genes in determining the final penetrance of the effect. Although it is difficult to provide a straightforward rationalisation for these differences, it is noteworthy that sv129 mice are more insulin sensitive than C57BL6 animals [113], with the latter producing more insulin in hyperglycemic clamps. It is possible, therefore, that C57BL6 mice are better equipped to tolerate perturbations in insulin storage and secretion following *ZnT8* deletion.

These data reinforce the idea that mice, at least, are able to adapt metabolically to the loss of ZnT8 alleles under many circumstances. However, under metabolic stress, such as in the case of a diet rich in fat, the impact of defective insulin storage and or secretion are more apparent at least for mice on a mixed genetic background.

Whilst complete inactivation of ZnT8 in the mouse has been useful as a means of understanding the function of this protein, it is clear that more work needs to be done in order to elucidate the significance of the diabetes-associated polymorphism *in vivo*. At present, knock-in models for either the protective (W325) or risk (R325) forms of ZnT8 are missing and may be revealing, provided that the impact on transporter activity is sufficiently large [97]. Of note, such models would more closely mimic the situation in humans and help us to better understand the metabolic, signaling, and other pathways that are altered in tissues which express the transporter.

## 4. Perspectives

### 4.1. Better Mouse Models

A key point to bear in mind in assessing the usefulness of mouse models is the relative plasticity displayed by rodents faced with gene deletions. Thus, differences between the penetrance of mutations in human genes linked to monogenic forms of diabetes, including maturity onset diabetes of the young (MODY), between humans and mice, are usually observed [114] with the mouse equivalents showing far less marked disturbances in glycemia or changes which are seen only after deletion of both alleles. This clearly reflects the limitations of the use of mice (weight ~25 g, life expectancy ~3 years) for comparisons with human subjects. Nonetheless, and although the phenotypes of the above murine models are thus often more subtle than the human counterparts, they remain useful models for the study of diabetes, allowing single-targeted gene deletions which are impossible in man. For example, human populations with different genetic backgrounds have different susceptibility to the R235W ZnT8 polymorphism. We should not, therefore, find surprising the results that different genetic backgrounds and different diet reveal different phenotypes in ZnT8 knockout models.

The study of knockout mouse models is most useful if the likely target gene is clearly defined, as is the case when a SNP lies in an exon and encodes a nonsense or missense mutation (as for *SLC30A8*). One of the difficulties in studying the contribution of the SNPs identified for increased risk of T2D is that many of the SNPs identified to date mainly reside in intronic regions. This may be due to the technical limitations of identifying the disease-causing gene using current methods for GWAS, or that the disease-inducing variation may indeed reside in the intronic region of the gene, which may have regulatory function, as may be the case for *TCF7L2* [85]. Frequently, the sequences within the SNP regions are poorly conserved between mouse and man, for example, the sequences spanning SNP rs7903146 for *TCF7L2* lies within a repetitive element that is absent in mice. One possibility is to conduct physiological studies in “humanized” mice [85], but it is difficult to fully replicate the human genetic environment in mouse models. Additionally, it is technically difficult to introduce targeted changes at high efficiency at precise locations. The emergence of genome modification technologies such as transcription activator-like effector nucleases (TALENs) [115–117] can substantially speed up the making of a tailored mutant animal model for whole system approaches to study the contribution of risk variants identified by GWAS to disease progression and may be useful in those instances where the region containing the variation is sufficiently similar to that found in humans. Importantly, such gene-editing approaches may also facilitate the use of alternative species (such as the rat or even the pig) whose physiology more closely resembles that of man.

An additional complication is that disease-causing SNPs do not exist in isolation. The genetic landscape of each individual may play a part in an individual's risk of developing a certain disease. For example, the risk of T2D is additive: the larger the number of risk SNPs present in an individual's genome, the higher the risk for the development of T2D [37, 118–121]. Thus, future animal models may require careful mapping of the genetic variations present in the model animal and the introduction of more than one genetic variation to model the diabetic phenotype conferred by these combined genetic variations.

### 4.2. Gene-Environment Interactions

An individual's risk of developing T2D is the product of interaction between the individual's genetic constitution and the environment inhabited by the individual. Whilst the contribution of genetic factors to disease risk is relatively easy to quantify, the impact of environmental exposure is less easily measured in a clinical setting. Nevertheless, efforts have been made to study the interactions between some of the known susceptibility *loci* for T2D and the environment, and these findings may be useful for the development of prediction models and tailoring clinical treatment for T2D [122, 123]. For example, for carriers of the risk allele for *TCF7L2*, diets of low glycaemic load [124, 125] and a more intensive lifestyle modification regime (*versus* that recommended for nonrisk carriers) [61, 62, 126, 127] have been shown to reduce the risk of T2D. Meaningful studies for gene-environment interactions will require samples of sufficient size to increase statistical power [128] and accurate methods for measuring environmental exposure, for example, the use of metabolomics to identify and assess metabolic characteristics, changes, and phenotypes in response to the environment,  diet, lifestyle, and pathophysiological states. This information will allow the generation of better risk prediction models and personalisation/stratification of treatment, the holy grail of GWAS.

### 4.3. Cancer versus Diabetes (Opposing Mechanisms Hypothesis)

One other observation from GWAS that should be mentioned, as it may have implications on treatment, is the link between cancer and T2D. There is epidemiological evidence that links T2D and cancer [129]. A large number of  T2D genes found via GWAS are involved in cell cycle regulation [34], for example, the T2D association SNP mapping to chromosome 9p21 in the vicinity of the tumour suppressor genes CDKN2A and CDKN2B [130–132] and the CDKN2B regulator ANRIL [133–135]. Recent genetic data suggest that common genetic variants influence cancer and diabetes in opposite directions [136, 137].

### 4.4. Which Genes Do We Study?

A fundamental challenge facing those wishing to determine which of the genes in a particular locus is responsible for affecting disease risk, and dissect how this/these act, is the very scale of the problem (currently more than 500 genes in total to interrogate, with others emerging) [35] (and McCarthy M, personal communication). Clearly, new strategies will be required both to prioritise genes and thus develop models for those most likely to be involved: assessment of the impact of a particular variant (odds ratio) as well as expression profile (notably expression in *β* cells for those genes affecting insulin secretion), and finally, the likely biological impact of variations in a particular gene based on published knowledge are all essential to this process. Further, “experimental filtration” through higher throughput approaches (e.g., siRNA in *β* cell lines, including novel human lines [138]) are likely to be needed. Finally, more high throughput means to inactivate or overexpress genes in specific tissues in living mice without the need to engineer the latter via conventional recombination-based engineering of embryonic stem cells (e.g., through virus-mediated delivery) [139] and are likely to be increasingly important. A further challenge is that of understanding how the identified genes affect disease risk work via different tissues; systems and computational biology are likely to be highly important here.

## Figures and Tables

**Table 1:** Summary of the major phenotype of the different colonies of ZnT8 KO mice. ZnT8-*α*KO and ZnT8-*β*KO for *α* and *β*-cell-specific knockout mice, respectively; GSIS for glucose-stimulated insulin secretion.

Phenotype/model	ZnT8KO-London [97]	ZnT8KO- Toronto [98]	ZnT8KO- Leuven [99]	ZnT8KO- Vanderbilt [100] (129SvEv^Brd^ × C57BL/6J)	ZnT8KO-Vanderbilt [101] (C57BL/6J)	ZnT8-*α*KO	ZnT8-*β*KO [102]
Glucose tolerance							
≤6 weeks	♂ intolerant	♂ intolerant ♀ intolerant	Normal		♂ intolerant	Normal	Intolerant
12 weeks	♂ intolerant ♀ normal	♂ normal ♀ intolerant	Normal				
≥18 weeks			Normal	Normal	Normal		

Insulin sensitivity	Normal	Normal	Normal	Normal	Normal		

Plasma glucose		♂: Elevated (fasting) at 6 wks, normal afterwards. ♀: Normal	Normal	Normal	Normal (fasting)		

Plasma insulin		Decreased	Normal	Decreased	Normal (fasting)	Normal. (Plasma glucagon normal.)	Normal

Islet insulin content			Normal (glucagon content was normal.)	Normal	Normal		

Insulin secretion							
*In vivo *		Reduced					
*In vitro *	Basal secretion enhanced GSIS normal	GSIS enhanced	GSIS normal	GSIS reduced	GSIS normal		Reduced first phase

Glucagon secretion		Unaffected					

Insulin processing	Normal		Normal				

Granule morphology	Abnormal	Abnormal	Abnormal		Normal	Normal	Abnormal
